# Engineered macrophages: an “Intelligent Repair” cellular machine for heart injury

**DOI:** 10.1186/s13619-024-00209-7

**Published:** 2024-11-27

**Authors:** Zhuo Zhang, Hetian Du, Weijie Gao, Donghui Zhang

**Affiliations:** 1 State Key Laboratory of Biocatalysis and Enzyme Engineering, School of Life Sciences, Hubei University, Wuhan, 430062 China; 2 Stem Cells and Tissue Engineering Manufacture Center, School of Life Sciences, Hubei University, Wuhan, 430062 China

**Keywords:** Engineered macrophage, Macrophage, Myocardial injury and repair, Inflammation, Targeted delivery, Gene editing

## Abstract

Macrophages are crucial in the heart’s development, function, and injury. As part of the innate immune system, they act as the first line of defense during cardiac injury and repair. After events such as myocardial infarction or myocarditis, numerous macrophages are recruited to the affected areas of the heart to clear dead cells and facilitate tissue repair. This review summarizes the roles of resident and recruited macrophages in developing cardiovascular diseases. We also describe how macrophage phenotypes dynamically change within the cardiovascular disease microenvironment, exhibiting distinct pro-inflammatory and anti-inflammatory functions. Recent studies reveal the values of targeting macrophages in cardiovascular diseases treatment and the novel bioengineering technologies facilitate engineered macrophages as a promising therapeutic strategy. Engineered macrophages have strong natural tropism and infiltration for cardiovascular diseases aiming to reduce inflammatory response, inhibit excessive fibrosis, restore heart function and promote heart regeneration. We also discuss recent studies highlighting therapeutic strategies and new approaches targeting engineered macrophages, which can aid in heart injury recovery.

## Background

Cardiovascular disease is the top leading cause of death worldwide, with a total prevalence of 48.6% among adults aged 20 and above (Martin et al. 2024; Tsao et al. 2023). The immune response is essential in cardiovascular disease development (Swirski and Nahrendorf 2018). Immune cells, especially macrophages, have strong chemotaxis and plasticity, making them excellent "seeds" for cell therapy (Na et al. 2023). Macrophages, as constituents of the mononuclear phagocyte system, participate in the development of cardiovascular diseases through accumulation in the damaged area. Monocytes and macrophages exert pro-inflammatory and anti-inflammatory effects on cardiovascular disease by switching to different phenotypes (Yap et al. 2023). Recently, engineered macrophages exhibit potent therapeutic value in treating cardiovascular diseases (He et al. 2020; Tan et al. 2024). Engineered macrophages are capable of recognizing and responding to distinct heart injury states, acting as a “living drug” when transferred into patients (Irvine et al. 2022). In this review, we discuss the multifaceted contributions of diverse macrophages in cardiac injury and the novel and currently available therapeutic strategies for treating cardiovascular diseases based on macrophages. Specifically, we discuss the new techniques and strategies for engineered macrophages and the prospects of their application in cardiovascular diseases.

## Macrophages in cardiac disease

### Resident and recruited macrophages in the heart

**Fig. 1:**
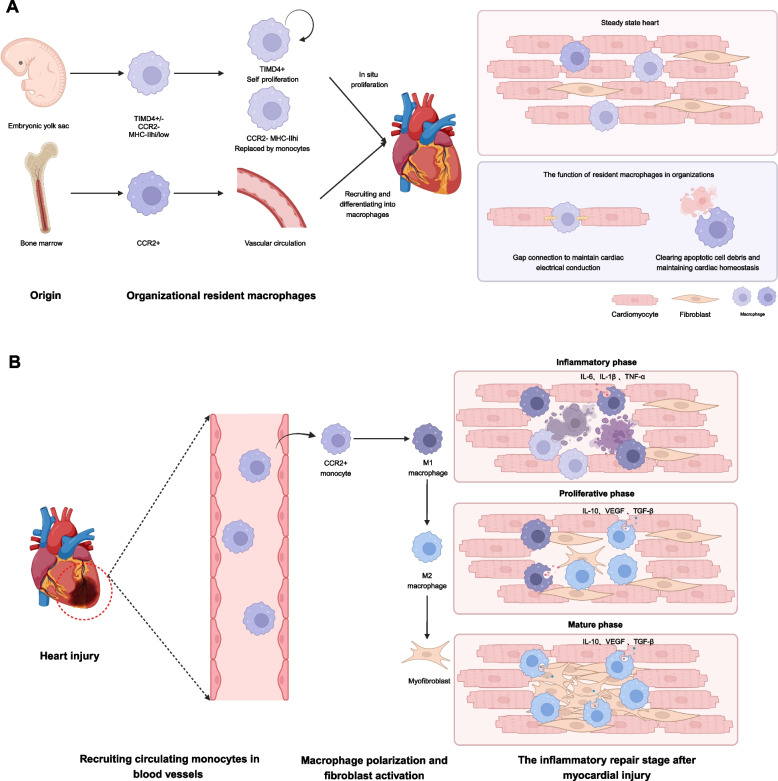
Different phenotypes and functions of macrophages in healthy and injured heart. **A** Cardiac macrophages are divided into two primary sources: 1) derived from the yolk sac stage of embryos, forming tissue-resident macrophages. Macrophages expressing TIMD4 undergo renewal through proliferation while circulating monocytes gradually replace macrophages that do not express CCR2; 2) Mononuclear cells originating from the bone marrow and circulating through blood vessels into the heart tissue. This cell type mainly expresses CCR2 and differentiates into macrophages after entering the heart. On the one hand, cardiac macrophages maintain electrical conduction by forming gap junctions with myocardial cells. On the other hand, it plays a role in phagocytosis and clearance, maintaining the homeostasis of the heart. **B** After heart injury, monocytes/macrophages circulating in the blood vessels are recruited. Differentiate into M1 proinflammatory macrophages in the damaged area, secreting inflammatory factors. As the inflammatory response subsides, M1 macrophages become M2 repair macrophages. M2 macrophages secrete cytokines such as TGF-β, which activate fibroblasts. Activated fibroblasts secrete extracellular matrix and participate in tissue remodeling

Macrophages are an inherent component of cardiac tissue, not only participating in maintaining normal cardiac homeostasis but also handling inflammatory responses and pathological progression. Cardiac macrophages can be divided into two categories: resident macrophages formed during the development of the heart and recruited macrophages derived from monocytes in peripheral blood, which migrate into the heart and differentiate into macrophages upon cardiac injury stimuli (Fig. 1A).

#### Origin of resident macrophages

Researchers examined the transcriptomes of various organs and identified three key gene regulatory networks in macrophages: (1) TLF^+^ (TIMD4^+^, LYVE1^+^, FOLR2^+^) for self-renewal; (2) CCR2^+^ (TIMD4^−^, LYVE1^−^, FOLR2^−^) replaced by monocytes; and (3) MHC-II^hi^ (TIMD4^+^, LYVE1^+^, FOLR2^+^, CCR2^−^) partially replaced by monocytes (Dick et al. 2022).

At E14.5, about 85% of yolk sac macrophages express TLF, lacking CCR2 and MHC-II. Single-cell analysis of the early fetal heart (5 to 9 PCW) showed most macrophages express LYVE1, FOLR2, and TIMD4 (Cui et al. 2019; Dick et al. 2022).

Lineage tracking revealed that heart macrophages mainly originate from the yolk sac and blood-derived endothelium of the aorta-gonad-mesonephric system (Liu et al. 2022). These macrophages are found in various locations, including the epicardium, endocardial cushion, outflow tract, and atrioventricular cushion (Gula and Ratajska 2022; Liu et al. 2022).

Researchers use a macrophage cell line with fluorescent markers to study their role in healthy mouse hearts (Hulsmans et al. 2017). In the atrioventricular (AV) node, macrophages interact with cardiomyocytes via the CX43 gap junction, enhancing myocardial cell repolarization and electrical conduction. Cardiac resident macrophages help maintain homeostasis through endocytosis; for example, Lgmn is specifically expressed by these macrophages, and its absence hinders their clearance function (Jia et al. 2022). Additionally, TREM2^hi^ CD163^+^ RETNLA^+^ macrophages can remove dysfunctional mitochondria from damaged myocardial cells (Zhang et al. 2023).

#### Recruitment of circulating monocytes and macrophages

Early studies indicate that monocytes generated from bone marrow circulate in the bloodstream and migrate into tissues, where they mature into macrophages (van Furth and Cohn 1968). Human peripheral blood monocytes can be classified into two groups based on surface proteins: CD14^hi^ CD16^−^ (classical monocytes) and CD14^+^ CD16^+^ (mature macrophages) (Gordon and Taylor 2005). In mice, monocyte subpopulations are identified by Ly6C expression; Ly6C^low^ monocytes are associated with the vascular system, whereas Ly6C^high^ monocytes are recruited to inflammatory sites (Honold and Nahrendorf 2018).

Damage-associated molecular patterns (DAMPs) from apoptotic cells are recognized by innate immune cells through toll-like receptors (TLRs), activating downstream signaling pathways (Arslan et al. 2011; Neu et al. 2022). MyD88 acts as a key molecule in this signaling (Bayer and Alcaide 2021). CCR2^+^ macrophages in damaged myocardial tissue induce chemokine expression (CCL2/MCP1 and CCL7/MCP3) via MyD88-dependent pathways, facilitating monocyte recruitment (Bajpai et al. 2019). Following cardiac injury, chemokines like CCL2 help recruit monocytes by binding to CCR2 (O'Connor et al. 2015).

### Polarization and activation of macrophages

During inflammation, macrophages respond to various signaling stimuli and are activated into either M1 or M2 types. M1 macrophages, which are pro-inflammatory, secrete cytokines such as IL-6, IL-1β, and TNF-α. In contrast, M2 macrophages are associated with resolving inflammation and aiding tissue repair by producing anti-inflammatory cytokines such as IL-10, VEGF, and TGF-β (Ambarus et al. 2012; Liu et al. 2014; Tarique et al. 2015).

M1 macrophages can be stimulated by IFN-γ and LPS. IFN-γ activates JAK1/JAK2 signaling by binding to the IFN-γ receptor (IFN-γR), which in turn activates STAT1 signaling molecules. This process promotes the expression of IL-12 and NOS2 in macrophages. LPS, when bound to TLR4, activates downstream NF-κB signaling pathways, leading to macrophage secretion of IL-6 and TNF-α.

M2 polarization typically involves cytokines like IL-4 or IL-13, which bind to the IL-4 receptor (IL-4Rα) on the cell surface. This interaction leads to the expression of Arg1 through STAT6 signaling. Additionally, it regulates IRF4, which promotes the expression of Arg1, Ym1, and Fizz1 (Lawrence and Natoli 2011; Liu et al. 2014). Furthermore, LPS binding to TLR4 enhances CREB-C/EBPβ signaling, which can also promote M2 macrophage development (Ruffell et al. 2009).

Recent findings show that macrophage classification is more complex than binary methods (Table 1) (Jung et al. 2022; Rizzo et al. 2023; Xu et al. 2023). Various specific subtypes have been identified, revealing their roles in cardiovascular disease, which could present potential therapeutic targets (Ma et al. 2018; Zaman et al. 2021).

**Table 1:** Macrophage markers during heart injury

Cell type	Marker gene	Ref
**Resident tissue macrophages**	Mφ1: Lyve1, F13a1, Cbr2, Cd163, Folr2, Timd4 Mφ2: H2-Eb1, H2-Aa, H2-Ab1, Cd74	Jung et al. (2022)
	Lyve1^+^ ResMφs: LYVE1 MHCII^±^ CCR2^−^ MHCII^+^ ResMφs: LYVE1^−^ MHCII CCR2^±^	Xu et al. (2023)
	RTM-TIMD4: TIMD4^+^ LYVE1^+^ MHCII^low^ Folr2^hi^, CD163, MGL2 and part expressed VSIG4 RTM-MHCII: MHCII, MGL2 and low expression of TIMD4	Rizzo et al. (2023)
**Pro-inflammatory macrophages**	Cd68, Fcgr1, Itgam, Ccr2	Jung et al. (2022)
	Il1b, Tnip3, Tlr2, Tnfsf9	Rizzo et al. (2023)
**Anti-inflammatory macrophages**	Mφ1: Trem2, Gpnmb, Fabp5, Spp1, Timp2 Mφ2: Apoe, Fcrls, Rgs10, Adgre1	Jung et al. (2022)
	Bhlhe41^hi^: Ctsd, Ccl8, Grn, Gpnmb	Xu et al. (2023)
	Trem2^hi^ Spp1^hi^: Hmox1, Arg1, Anxa1 Trem2^hi^ Gdf15^hi^: Igf1, Gdf15, Mertk, Timp2, Apoe Trem2^hi^ Prdx1^hi^: Ftl1, Fth1, Slc40a1, Slc48a1	Rizzo et al. (2023)
**Proliferating macrophages**	Mφ1: Mcm6, Hells Mφ2: Top2a, Mki67	Jung et al. (2022)
	Stmn1, Top2a, Mki67, Birc5	Xu et al. (2023)
**IFN macrophages**	Irf7, Isg15, Ifit2	Jung et al. (2022)
	Isg15, Irf7, Cxcl10	Rizzo et al. (2023)

### Role of macrophages in cardiac disease

Cardiovascular disease is the leading cause of death globally. Macrophages, the primary innate immune cells, play a vital role in these conditions (Fig. 1B). After heart damage, necrotic cells release damage-associated molecular patterns (DAMPs) that attract circulating monocytes to the injured area. These monocytes replace resident macrophages and differentiate into pro-inflammatory macrophages, which secrete inflammatory factors and clear debris.

As inflammation progresses, pro-inflammatory macrophages transition into M2 macrophages, releasing TGF-β to activate fibroblasts. These activated fibroblasts, or myofibroblasts, then secrete extracellular matrix (ECM) components to aid in tissue repair (O'Rourke et al. 2019).

#### Infiltrated macrophages in myocarditis

Myocarditis is characterized by inflammation of the heart, primarily due to extensive infiltration of inflammatory macrophages (Sharma et al. 2019). Common causes include viral infections (like the Coxsackie virus), autoimmune diseases, immune checkpoint inhibitors, and vaccines (Ammirati and Moslehi 2023). This condition can be classified into explosive and non-explosive forms. Acute non-fulminant myocarditis may present unclear initial symptoms, progressing to chronic dilated cardiomyopathy and, in severe cases, end-stage heart failure. In contrast, fulminant myocarditis leads to sudden cardiac inflammation and cardiogenic shock (Kociol et al. 2020; Tschope et al. 2021).

Diagnosis typically involves an endocardial biopsy (Sharma et al. 2019), and macrophages in affected patients generally express CD68 (Goldman et al. 2022). Notably, macrophages, rather than T cells, predominantly express IL-7Rα, which regulates their transport and may present a new treatment target (Kubin et al. 2020).In immune checkpoint inhibitor (ICI) myocarditis, an explosion of CCR2^+^ monocyte-derived macrophages and CD8^+^ T cells occurs, with signaling pathways like IFN-γ and CXCR3 playing key roles (Ma et al. 2024).

In CVB3-induced myocarditis, the absence of Stabilin-1 affects monocyte recruitment, causing a pro-inflammatory environment and increased mortality (Carai et al. 2022). Reducing CAPN4 expression in macrophages can alleviate inflammation following CVB3 infection (Wang et al. 2023a, b, c, d). Additionally, SARS-CoV-2, responsible for COVID-19, can also trigger an inflammatory storm, affecting myeloid cells and IL-6 levels (Wang et al. 2023a, b, c, d).

#### Macrophage phenotype transition in myocardial infarction

Macrophages are essential for managing inflammation and tissue repair after a myocardial infarction (MI), which is a leading cause of death globally (Vaduganathan et al. 2022). MI occurs in three stages: the inflammatory phase (0~3 days), the proliferative phase (3~14 days), and the mature phase (lasting weeks) (Li et al. 2022).

Initially, resident macrophages in the heart produce inflammatory factors like IL-1 and TNF (Mouton et al. 2018). Within 24 h, circulating monocytes infiltrate the area, polarizing into pro-inflammatory types (Il1b, Mmp8). By day three, pro-inflammatory gene expression decreases, and macrophages’ phagocytic abilities increase (Mouton et al. 2018). By day seven, macrophages shift to a repair phenotype, promoting collagen deposition and angiogenesis, while continuing to downregulate inflammation (Mouton et al. 2018; Swirski and Nahrendorf 2018).

In Bajpai’s study, different macrophage types were shown to have distinct roles after cardiac injury (Bajpai et al. 2019). Recruited CCR2^+^ macrophages produce more inflammatory cytokines, while resident CCR2^−^ macrophages express growth factors and DNA repair genes (Revelo et al. 2021). The absence of resident macrophages can hinder cardiac remodeling processes (Dick et al. 2019).Rizzo’s team categorized macrophages in the MI area into pro-inflammatory (Isg15^hi^ and MHCII^+^ Il1b^+^) and a non-inflammatory group (Trem2^hi^), with the latter being linked to phagocytic function (Rizzo et al. 2023).

#### Pro-inflammatory macrophages in heart failure

Macrophages and inflammatory factors are key players in the development of heart failure, which affects about 26 million people worldwide and presents a significant public health challenge. Often the final stage of various cardiovascular diseases, heart failure has a five-year survival rate of less than 50% (Savarese et al. 2023).

CCR2^+^ macrophages are linked to left ventricular dysfunction, suggesting that blocking CCR2 may be a potential treatment (Bajpai et al. 2018). Pro-inflammatory cytokines such as TNF-α, IL-1β, and IL-6 contribute to myocardial hypertrophy and dysfunction (Shirazi et al. 2017). Inflammatory macrophages cause mitochondrial oxidative stress in myocardial cells by releasing IL-1β, ultimately leading to cardiac diastolic dysfunction. Using IL-1β receptor antagonist can be a potential HF therapeutic option (Liu et al. 2023). High IL-6 levels may serve as a biomarker for heart failure (Chia et al. 2021).

The soluble ST2 protein (sST2) is another important marker, and its combined use with natriuretic peptides can aid in managing heart failure patients (Aimo et al. 2019). IL-33 has both pro-inflammatory and cardioprotective effects depending on its binding to ST2 (Seki et al. 2009). While IL-10, upregulated in cardiac macrophages, promotes fibrosis. Deficiency of IL-10 in macrophages may improve diastolic function (Hulsmans et al. 2018).

In conclusion, inflammatory factors are critical not only as biomarkers for heart failure but also as potential therapeutic targets.

## Macrophage-targeted therapies for cardiac injury

Macrophages are vital during cardiac injury, as they help regulate inflammation and restore heart function. Their diversity allows for targeted therapies that can focus on specific macrophage subpopulations. This approach could lead to new treatments for cardiovascular diseases. We highlight three potential options: delivering macrophages, regulating their phenotype, and enhancing electrical conduction (Table 2).

**Table 2:** Summary of the strategy of macrophage therapy for myocardial injury

Strategy	Cell type	Species	Machenism	Effect	Ref
**Cell therapy**	Primary cells	Mouse	Nanoparticles loaded with Tβ4	Cell proliferation ↑	Chen et al. (2023)
	Primary cells	Mouse	Over-express VEGF	Angiogenesis ↑	Yan et al. (2013)
	Primary cells	Mouse	Transplant cardiac macrophages	Cardiomyocytes proliferation ↑	Li et al. (2021)
	Cell line	Mouse	Platelet membrane nanocarriers	Repair phenotype and angiogenesis↑	Xu et al. (2024)
	Cell line	Mouse	VEGF-nanoparticles coated with NAC-modified macrophage membrane	M2 phenotype polarization, antioxidant, and blood vessels formation ↑	Zhu et al. (2024)
	Engineered macrophages cells	Mouse, zebrafish	Gene editing and transplant macrophages	Fibrosis ↓	Simoes et al. (2020)
**Regulate phenotype transition**	Primary cells	Mouse	Activate TGF-β/Smad signaling	Repair phenotype and phagocytosis ↑	Chen et al. (2019)
	Primary cells	Mouse	Mertk resistant to cleavage	Phagocytosis and anti-inflammatory ↑	DeBerge et al. (2017)
	Primary cells	Mouse	Inhibit AXL	Inflammatory phenotype ↑	DeBerge et al. (2021)
	Primary cells	Mouse	Biomimetic nanomaterials inhibit CD47-SIRPα axis	Phagocytosis ↑	Gao et al. (2024a, b)
	Primary cells	Mouse	Mitochondrial targeted antioxidant	Endocytosis ↑	Cai et al. (2023)
	Primary cells	Mouse	Inject soluble Trem2	Phagocytosis	Jung et al. (2022)
	Primary cells and cell line	Mouse, human	cGAS functional inhibition	M2 macrophages and blood vessels formation ↑, fibrosis ↓	Cao et al. (2018)
	Engineered macrophages cells	Mouse	Over-express CCR2 and cleavage-resistant MerTK, liposomal PEP-20	Endocytosis and anti-inflammatory ↑	Tan et al. (2024)
**Restore electrical conductivity**	Primary cells	Mouse	CX43 gap connection	Cardiac conduction ↑	Hulsmans et al. (2017)
	Primary cells	Mouse	Amphiregulin (AREG)	Gap junctions ↑	Sugita et al. (2021)
	Primary cells	Mouse	Knockout IL-1 β	Electrical remodeling ↑	Sun et al. (2016)
	Primary cells	Rat	SDF-1α-encapsulated Puerarin (PUE) hydrogel	CX 43 ↑, and electrical conductivity ↑	Luo et al. (2024)
	Cell line	Human	Glucocorticoids	Anti-inflammatory ↑	Hutschalik et al. (2024)

### Macrophage-based cell therapy

Recent studies have shown that CD68-GFP macrophages are recruited to the collagen deposition sites following the adoptive transfer of CD68-GFP labeled adult monocytes into injured hearts (Simoes et al. 2020). Furthermore, transplanting neonatal murine cardiac macrophages can enhance cardiac repair in adult myocardial infarction by promoting myocardial cell proliferation (Y. Li et al. 2021). In experiments where engineered macrophages overexpressing VEGF were injected into mice with myocardial infarction, these VEGF^+^ macrophages migrated to the blood vessels in the injury boundary area and contributed to angiogenesis (Yan et al. 2013). Additionally, macrophages possess distinctive inflammatory chemotaxis and targeted recognition mechanisms, making them potential carriers for drug delivery. By utilizing nanotechnology to load VEGF onto macrophage membranes, these cells can accumulate in the myocardial infarction area through their chemotactic properties, thereby releasing VEGF to promote angiogenesis (Zhu et al. 2024).

On one hand, the CD47 protein, known as the "do not eat me" protein, is expressed on the surface of macrophages, allowing them to evade detection by the body’s immune system. On the other hand, macrophages recruited after injury express high levels of CCR2 receptors, which help them target and recognize damaged sites. Therefore, binding extracellular vesicles derived from monocyte membranes (loaded with drugs) to circulating macrophage membranes can enable immune escape and targeted localization at damaged areas (Chen et al. 2023). Additionally, CD62P expressed on platelet membranes has a strong affinity for macrophages, particularly for circulating Ly6C^+^ monocytes. The adhesion of platelet membranes during inflammation activates macrophages, allowing them to target the damaged areas of the heart, thereby reducing off-target effects and achieving more efficient and accurate drug delivery (Xu et al. 2024).

### Regulating macrophages phenotype transition

The transformation of macrophages from inflammatory to tissue repair phenotypes is crucial for healing. When the heart is damaged, the DNA sensor cGAS is activated, triggering the STING cascade and promoting M1 macrophage activation. Inhibiting this pathway can enhance M2 macrophage activation, improving tissue repair and survival rates after myocardial infarction (Cao et al. 2018).

After three days of heart damage, IL-10, Hif1a, and Stat1 increase, promoting a transition from pro-inflammatory to anti-inflammatory macrophages (Walter et al. 2018). TGF-β activates Smad3 signaling, mediating phagocytic phenotype acquisition and protecting the infarcted heart (Chen et al. 2019). MerTK, expressed by macrophages, plays a key role in clearing apoptotic cells and regulating inflammatory cytokines. Lack of MerTK hinders heart recovery (DeBerge et al. 2017). Researchers have developed macrophages that over-express CCR2 and cleavage-resistant MerTK (MerTKCR), along with CD47 antagonists, to enhance phagocytic function (Tan et al. 2024). AXL also affects macrophage metabolism and inflammatory phenotype. Maintaining a balance of AXL is vital for cardiac recovery (DeBerge et al. 2021). Blocking the CD47-SIRPα pathway enhances macrophage phagocytosis of apoptotic cardiomyocytes after myocardial infarction (Gao et al. 2024a, b). Myeloid-specific deletion of the mitochondrial protein encoded by Ndufs4 impairs macrophage phagocytosis and delays tissue repair (Cai et al. 2023). TREM2^hi^ macrophages have anti-inflammatory properties, and their overexpression or injection can significantly improve cardiac function post-infarction (Gong et al. 2024; Jung et al. 2022; Rizzo et al. 2023).

### Restore electrical conductivity

Cardiac macrophages are essential for maintaining electrical conduction in the heart by forming gap junctions with myocardial cells via the protein CX43. A lack of CX43 in macrophages or congenital macrophage deficiency can delay atrioventricular conduction (Hulsmans et al. 2017). These macrophages can also depolarize the resting membrane potential of myocardial cells and influence action potential duration (Simon-Chica et al. 2022).

The production of amphiregulin (AREG) by cardiac macrophages is crucial for regulating CX43. Its absence can lead to abnormal electrical conduction and arrhythmias, as seen in patients with sudden cardiac death (Son et al. 2014; Sugita et al. 2021). Pro-inflammatory macrophages accumulate in the atria of patients with atrial fibrillation, secreting IL-1β, which inhibits QKI expression in cardiomyocytes. Knocking out IL-1β can restore QKI levels (Sun et al. 2016). In myocardial infarction animal models, increased KCNN4 expression in MI macrophages leads to prolonged action potential duration. Recruiting M2 macrophages can mitigate excessive inflammation and improve electrical conductivity (Luo et al. 2024).

Co-culturing human embryonic stem cell (hESC) macrophages with engineered myocardial tissue can enhance its functional maturation (Hamidzada et al. 2024). However, pro-inflammatory macrophages can disrupt cardiomyocytes’ regular beating, contributing to arrhythmias (Hutschalik et al. 2024). Understanding these interactions is crucial for strategies to maintain cardiac conduction and prevent arrhythmias.

## Engineered macrophages for cell therapy in cardiac injury

Cell therapy is a promising treatment approach that offers distinct clinical and therapeutic advantages over traditional drug therapies. Living cells can perform complex biological functions and essential cellular processes dynamically. Numerous cell therapy products have already received approval and are currently being investigated in clinical research, indicating broad application prospects (Wang et al. 2021).

### MSC and CAR-T cell therapy for myocardial injury

Mesenchymal stem cells (MSCs) are easy to obtain, can expand in vitro, and have low immunogenicity. They can differentiate into cardiomyocytes, and transplanting cell patches from human umbilical cord MSCs can improve inflammation and cell survival in infarcted hearts (Guo et al. 2021; Guo et al. 2020). However, clinical use still faces challenges like poor migration and low survival rates. Engineered human mesenchymal progenitor cells (FOXO3-GE-MPCs) can enhance healing after myocardial infarction, with the FOXO3 gene extending their survival (Lei et al. 2021).

Chimeric Antigen Receptor (CAR) technology enables immune cells to specifically recognize and attack target cells. CAR-T cell therapy has shown success in treating cancers but can also face limitations, including cardiac toxicity (Lefebvre et al. 2020; Rurik et al. 2022).

### Technology of engineered macrophage for myocardial injury

**Fig. 2:**
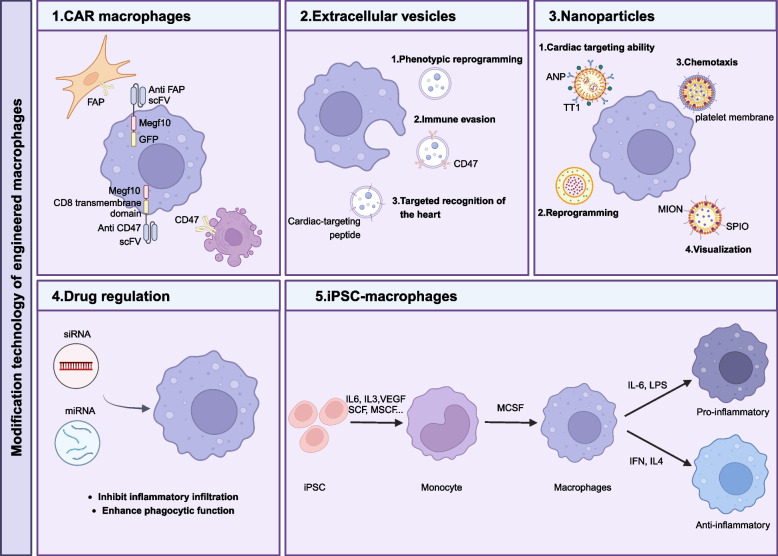
Technologies for transforming engineered macrophages. The techniques for obtaining engineered macrophages in vitro can be divided into CAR-macrophages, extracellular vesicles, nanoparticles, drug regulation and iPSC-derived macrophages

Cell engineering entails the genetic or non-genetic modification of cells to improve their targeting abilities and functional efficiency. The modified cells are subsequently re-injected into the patient’s body to address specific diseases. Modifying macrophages to create engineered macrophages has opened new avenues for treating cardiovascular diseases. In this section, we summarize the specific techniques used to produce engineered macrophages (Fig. 2).

#### CAR macrophages

CAR-M therapy involves transferring the CAR gene into macrophages, enhancing their ability to phagocytose target cells by binding to specific antigens. Compared to CAR-T cells, CAR macrophages (CAR-Ms) have advantages, including better tissue infiltration and reduced cytotoxicity (Hu et al. 2023). Unlike CAR-T cells, macrophages can easily penetrate complex extracellular matrices and have a limited lifespan, minimizing excessive immune responses.

Chimeric antigen receptor phagocytes (CAR-P) boost phagocytic activity by expressing the intracellular domain of MegF10 or FcRv. Gao’s team developed a CAR-P targeting FAP^+^ fibroblasts, utilizing a scFv, a cytoplasmic domain, and a GFP tag. The BMDM CAR-P effectively targets myofibroblasts and reduces fibrosis in a heart injury model while not affecting long-term survival (Gao et al. 2024a, b).

However, CAR-M therapy has limitations. Injected macrophages do not proliferate within the host, requiring careful control of cell numbers. Additionally, monitoring off-target effects is essential to prevent unwanted infiltration into healthy organs. Research has shown CAR-Ms can target and clear CD47-expressing apoptotic cells, improving debris clearance and reducing inflammation (Chuang et al. 2024).

While CAR-M has shown promise in animal models, more in vivo studies are needed to assess its effectiveness and potential side effects. Future efforts will focus on developing additional phagocytic targets for damaged hearts and combining therapies for enhanced treatment outcomes.

#### Extracellular vesicles

Extracellular vesicles (EVs) are nanoscale structures produced via endocytosis, known for their biocompatibility and ability to penetrate biofilms. They contain bioactive molecules like proteins, DNA, and RNA and are also produced by macrophages.

Recent studies demonstrate that small EVs from M2 macrophages, when injected into hearts damaged by ischemia–reperfusion (I/R), can promote anti-inflammatory activity and aid in cardiac repair (Li et al. 2023). However, delivering EVs to the heart is challenging, as those from external sources are often cleared by circulating monocytes and macrophages (Chen et al. 2021).

To enhance targeting, researchers found that binding EVs derived from monocyte membranes expressing CD47 to circulating macrophage membranes can improve localization to damaged areas (Tan et al. 2024). Additionally, CTP/PM-M2 EVs^vMIP−II−Lamp2b^, enriched with viral macrophage inflammatory protein-II (vMIP-II), a cardiac-targeting peptide (CTP), and platelet membrane (PM), enhances targeting to the heart and regulates inflammatory macrophages in viral myocarditis (Pei et al. 2024).

EVs are typically isolated through ultracentrifugation and chromatography. For therapeutic use, obtaining high-purity EVs is essential, as mixed sources can affect treatment outcomes. Exploring engineering methods for EV acquisition could provide new solutions, while considering the dual regulatory role between EVs and macrophages.

#### Nanoparticles

Nanoparticles enhance drug stability and reduce off-target effects. They have potential for targeting macrophages, as the immune system, especially macrophages, can recognize them and induce polarization.

Acetylated dextran (AcDEX) nanoparticles have been modified with ANP and TT1 peptides for targeting cardiac cells and macrophages related to atherosclerotic plaque, with M2-like macrophages showing higher binding capacity (Torrieri et al. 2020). Combining neutrophil apoptotic bodies with mesoporous silica nanoparticles (MSN) loaded with HAL effectively reprograms macrophages in the infarcted area, aiding in inflammation regulation (Bao et al. 2022). SiRNA targeting S100A9 coated with modified macrophage membrane can target damaged myocardial cells and weaken lysosomal ablation (Lu et al. 2024).

Phosphatidylserine (PS) binds to PS receptors on macrophages, promoting the transformation from M1 to M2 macrophages. M2 macrophages secrete TGF-β1 and IL-10 to aid blood flow restoration and healing. Magnetic iron oxide nanocubes (MIONs) enhance PS retention through magnetic targeting and can be visualized using MRI (Chen et al. 2017). Macrophage membrane-modified nanoprobes show low immunogenicity and direct inflammation-oriented chemotaxis (Yin et al. 2024). Therefore, nanotechnology holds promise as a novel clinical diagnostic tool (Wang et al. 2023a, b, c, d). However, it is crucial to assess the potential toxic effects of accumulated nanoparticles, which could impede tissue recovery.

#### Drug regulation

Targeting myocardial macrophages is a promising strategy for treating cardiovascular diseases. Biomolecules like siRNA and miRNA can serve as therapeutic agents by manipulating signaling pathways. MiRNA-21 is known for its anti-inflammatory effects, inhibiting cell infiltration (Yang et al. 2018). MiRNA-21 mimetics, delivered via nanoparticles, can shift macrophages from a pro-inflammatory to a reparative phenotype, promoting angiogenesis and reducing hypertrophy, fibrosis, and apoptosis (Bejerano et al. 2018).

In another study, encapsulated MOF-siRNA (siMOF) and microRNA-21 (miR21) released into myocardial macrophages led to gene silencing, decreased expression of MOF and KBTBD7 genes, and inhibited the NFkB signaling pathway (Wang et al. 2022). M2 macrophage-derived exosomes can release miRNA-1271-5p to regulate SOX6, decreasing hypoxia-induced cardiomyocyte apoptosis and improving viability (Long et al. 2021). Additionally, miR-149 targets CD47 to promote phagocytosis (Ye et al. 2019).

For effective clinical application, it's essential to design delivery systems that enhance the stability of miRNA/siRNA in vivo, potentially by combining nanoparticles with extracellular vesicle technology.

#### iPSC-derived macrophages

Induced pluripotent stem cells (iPSCs) can proliferate indefinitely and be directed to differentiate into macrophages, providing a valuable source for research and therapy. Studies show that these macrophages can adopt different phenotypes, with M2 macrophages alleviating liver fibrosis in mice (Pouyanfard et al. 2021).

Human iPSCs (hiPSCs) can produce monocytes and macrophages that closely resemble those from peripheral blood, displaying higher levels of phagocytic and endocytic-related genes (X. Cao et al. 2019). Additionally, embryonic stem cells can generate LYVE1^+^ macrophages that mitigate myocardial stress by clearing apoptotic cells (Hamidzada et al. 2024). These differentiated macrophages also contribute to angiogenesis in engineered heart tissue models (Landau et al. 2024).

Furthermore, CAR-expressing macrophages can be derived from iPSCs (Wang et al. 2023a, b, c, d). Overall, iPSC-derived macrophages offer a promising platform for treating cardiovascular diseases.

### Engineered macrophages for cardiovascular disease treatment

**Fig. 3:**
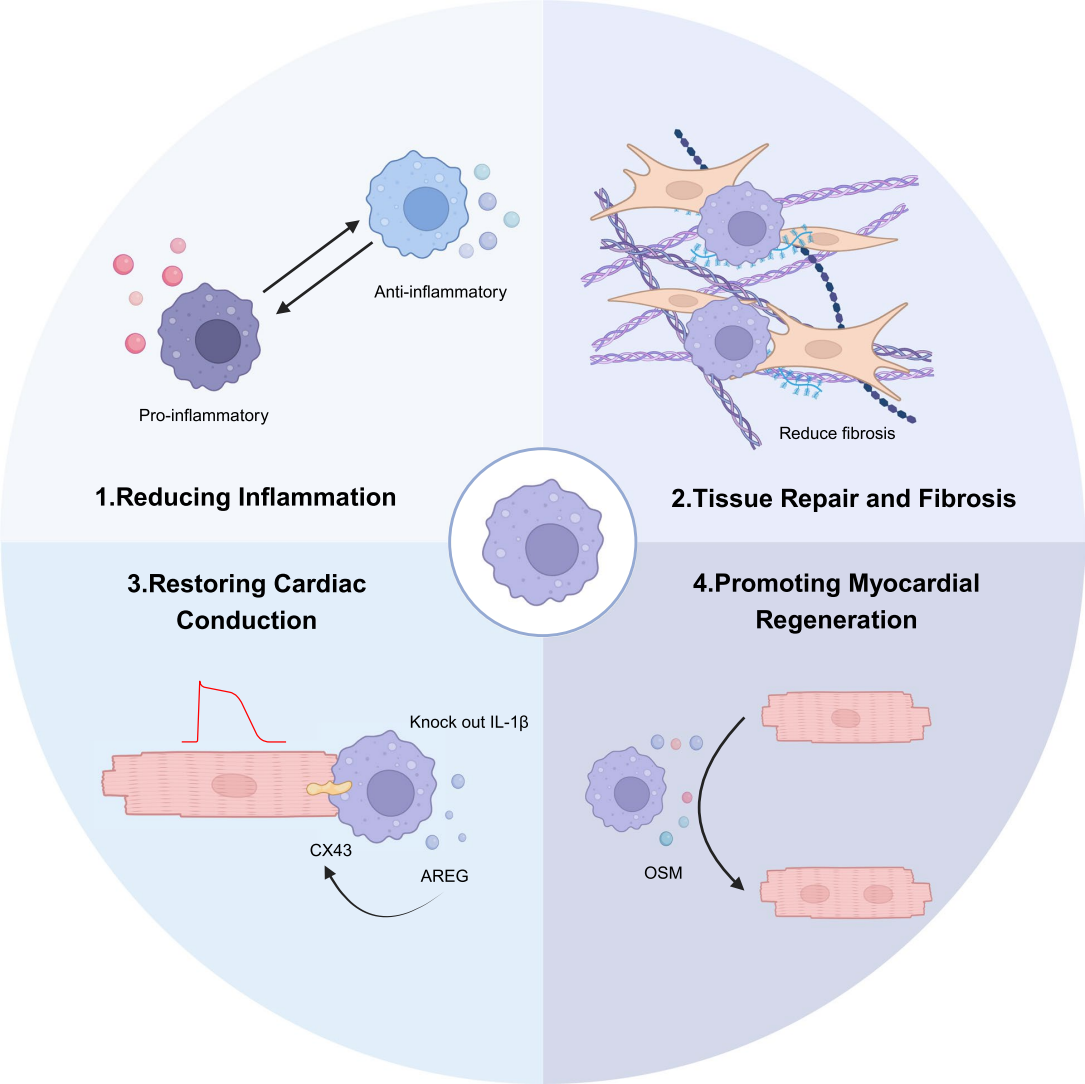
Strategies for engineered macrophages treatment. The strategy of treating myocardial injury by engineered macrophages can be considered various aspects, like anti-inflammatory, fibrosis reduction, restoration of heart function, and myocardial regeneration

The strategy for engineered macrophages to treat cardiovascular diseases focuses on four main areas: reducing inflammation, inhibiting fibrosis, restoring cardiac function, and promoting heart regeneration (Fig. 3).

#### Reducing inflammation

Chronic inflammation can damage tissue and hinder healing. Using siRNA to silence specific genes in macrophages can shift them from a pro-inflammatory to a reparative state, reducing reactive oxygen species (ROS) and inflammatory factors (Wang et al. 2022). Knocking out macrophage-specific Lgr4 decreases inflammatory cells in the injury area, reduces myocarditis, promotes angiogenesis, and improves cardiac function (Huang et al. 2020).

#### Tissue repair and fibrosis

Macrophages are essential for tissue repair, but excessive fibrosis can impair heart function. In zebrafish, pro-inflammatory macrophages (TNF-α^+^) promote scar deposition, while non-inflammatory macrophages help in scar removal (Bevan et al. 2020). A knockout model shows reduced scarring in damaged hearts (Simoes et al. 2020).

#### Restoring cardiac conduction

Myocardial dysfunction can lead to arrhythmias. Macrophages contribute to cardiac conduction, with AREG from resident macrophages being crucial for this process (Sugita et al. 2021). Knocking out IL-1β in macrophages restores disrupted cardiomyocyte function (Sun et al. 2016).

#### Promoting myocardial regeneration

Adult myocardial cells are limited in their regenerative capacity. Macrophages promote their proliferation through Oncostatin M (OSM) (Li et al. 2020). They also stimulate epicardial cell proliferation, which aids in myocardial regeneration (Bruton et al. 2022). Balancing macrophage phenotypes is key to scar regression and complete cardiac regeneration (Bevan et al. 2020; Simoes et al. 2020).

## Conclusions and perspectives

This review highlights the role of macrophages in myocardial injury and their potential for repair. Their natural ability to migrate towards damaged areas makes them effective drug carriers for targeted release. Different macrophage phenotypes play specific roles in injury repair, and genetic modifications can enhance their targeting and migration efficiency.

Recent studies have shown that resident macrophages can connect with cardiomyocytes, affecting their communication and action potentials. Emerging macrophage cell therapies utilize techniques like CAR-M technology to improve phagocytosis and deploy extracellular vesicles that maintain low immunogenicity. However, engineered macrophages still have may limitations such as potential off-target effects and accumulation in unwanted organs.

Creating high-quality, single-phenotype engineered macrophages at scale is crucial. Besides using patient-derived monocytes, iPSC technology can yield patient-specific macrophages for personalized treatment. Transitioning from research to clinical application involves addressing safety concerns regarding survival, function, and off-target effects.

In conclusion, engineered macrophages offer a promising strategy for treating myocardial injury, integrating insights from cardiology, immunology, and materials science to achieve targeted tissue repair and restore cardiac function.

## Data Availability

Not applicable.

## Abbreviations

CAR: Chimeric antigen receptor
CCR2: Chemokine receptor C–C motif chemokine receptor 2
cGAS: Cyclic GMP-AMP synthase
CVB3: Coxsackievirus B3
CX43: Connexin 43
DAMPs: Damage associated molecular patterns
ECM: Extracellular matrix
EVs: Extracellular vesicles
hESC: Human embryonic stem cells
HF: Heart failure
ICI: Immune checkpoint inhibitor
MI: Myocardial infarction
MHC-II: Major histocompatibility complex II
MPCs: Mesenchymal progenitor cells
MSCs: Mesenchymal stem cells
OSM: Oncostatin M
PCW: Post-conception week
PRRs: Pattern recognition receptors
ROS: Reactive oxygen species
sST2: Soluble isoform of ST2
TLRs: Toll like receptors
